# Hospital Drain Microbiomes as Distinct Microbial Signatures Within Urban Wastewater Networks in the Han River Watershed

**DOI:** 10.3390/antibiotics15101041

**Published:** 2026-10-21

**Authors:** Md Sarower Hossen Shuvo, Won Ju-young, Sujin Jo, Izaz Ahmed, Md Tareque Aziz, Ali Atashi, Md Abu Raihan, Park Yeji, Cheong Hyunhee, Taehyeon Kim, Hyein Yu, Haneol Bae, Sukyung Kim, Hoonhee Seo, Ho-Yeon Song

**Affiliations:** 1 Department of Microbiology and Immunology, School of Medicine, Soonchunhyang University, Cheonan 31151, Republic of Korea; 2 K-Microbiome Institute, 813-26 Yisunsin-daero, Asan 31462, Republic of Korea; 3 Korea Total Healthcare Ecosystem Association (KoTHEA), Gukhoe-daero, Yeongdeungpo, Seoul 07238, Republic of Korea; wjo9956@gmail.com (W.J.-y.); peterparker9619@naver.com (H.B.)

**Keywords:** environmental surveillance, hospital drain microbiome, urban wastewater, microbial diversity, metagenomic profiling

## Abstract

**Background:** Hospital drainage systems represent an important interface between healthcare facilities and urban wastewater networks and may contribute distinct microbial inputs to downstream aquatic environments. This issue is particularly relevant in Seoul, Republic of Korea, where urban wastewater discharges into receiving waters within the Han River watershed. **Methods:** In this study, we characterized hospital drain microbiomes to evaluate their microbial and environmental signatures, using residential drains as a non-clinical comparison. **Results:** Hospital drains exhibited a distinct taxonomic profile, with greater relative abundances of *Bacteroidota* and *Firmicutes* and several genera of environmental or potential public health relevance, including *Mycobacterium*, *Legionella*, and *Pseudomonas*. Although microbial richness was comparable between the two environments, Simpson diversity was significantly lower in hospital drains, indicating reduced community evenness. Bray–Curtis and Jaccard analyses showed clear separation between hospital and residential microbial communities, and hierarchical clustering further supported environment-associated community structuring. LEfSe analysis identified distinct hospital-associated taxonomic biomarkers, and predicted functional profiling revealed substantial metabolic differentiation, with 19 of the 25 most significantly differentiated pathways enriched in hospital samples. **Conclusions:** These microbial and functional differences underscore the ecological uniqueness of hospital drains and support their inclusion in future wastewater-based microbial surveillance strategies. Collectively, these findings establish hospital drains as distinct microbial ecosystems within urban wastewater networks and provide a baseline for future studies examining the persistence of hospital-associated microbial signatures within the Han River watershed.

## 1. Introduction

Urban drainage systems represent important interfaces between human activities, wastewater infrastructure, and receiving aquatic environments, collecting microorganisms, organic matter, and chemical residues that support complex microbial communities [1]. Drain-associated microorganisms can form persistent biofilms, allowing drainage networks to act as reservoirs and transport pathways within the urban environment [2]. Among these systems, hospital drains deserve particular attention because they are exposed to healthcare-associated microbial and chemical inputs, including human-associated microorganisms, pharmaceuticals, antimicrobial compounds, and disinfectants [3]. These conditions may impose selective pressures that distinguish hospital drain microbiomes from residential drainage systems. Previous studies have shown that hospital plumbing and wastewater can harbor diverse microbial populations, opportunistic taxa, and antimicrobial-resistance determinants [4]. Therefore, profiling hospital drain microbiomes can provide valuable insights into healthcare-associated microbial signatures entering urban wastewater networks and support environmental microbial surveillance.

Microorganisms and microbial genetic material released through hospital drainage systems enter larger wastewater networks, where they may interact with microorganisms derived from residential and other urban sources [4]. Wastewater treatment can substantially reduce microbial loads; however, previous studies have shown that some microorganisms, antimicrobial-resistance genes, and other wastewater-associated contaminants may persist through different stages of treatment and subsequently occur in receiving aquatic environments [5]. These contaminants may disrupt aquatic microbial communities, promote antimicrobial-resistance dissemination, and increase exposure to opportunistic pathogens, while residual pharmaceuticals and disinfectants may impose additional ecological pressures [6]. This issue is particularly important in Seoul, Republic of Korea, where the Han River represents a major aquatic system within a highly urbanized metropolitan region. The city collects and processes wastewater through urban wastewater infrastructure associated with the Han River watershed. Characterizing hospital drains can therefore provide an upstream baseline for future studies examining whether healthcare-associated microbial signatures persist through wastewater treatment and become detectable in receiving waters. The present study does not directly trace hospital drainage to the Han River; rather, the Han River watershed provides the broader environmental context for evaluating the potential downstream relevance of hospital-associated microbial inputs.

To place hospital-associated microbial characteristics in an appropriate environmental context, we included residential drains as a non-clinical comparison. Residential drains experience many of the same fundamental ecological conditions as hospital drains, including intermittent water exposure, nutrient input, moist surfaces, and biofilm formation. However, domestic activities rather than healthcare practices primarily influence them [7]. This comparison helps identify taxonomic and ecological characteristics associated with hospital drainage while partially controlling for microbial features broadly shared by urban plumbing environments. Nevertheless, differences in microbial communities between hospital-associated and residential roadside drains remain poorly understood.

This study primarily aimed to characterize the microbial communities of hospital drains and evaluate their environmental significance within Seoul’s urban wastewater context, using residential drains as a non-clinical comparative group. This integrated approach establishes a baseline characterization of hospital-associated drain microbiomes. It provides a framework for future investigations tracing healthcare-associated microbial signatures through wastewater-treatment systems and into receiving aquatic environments, including the Han River.

## 2. Results

### 2.1. Distinct Microbial Signatures Characterize Hospital Drain Communities

**Figure 1:**
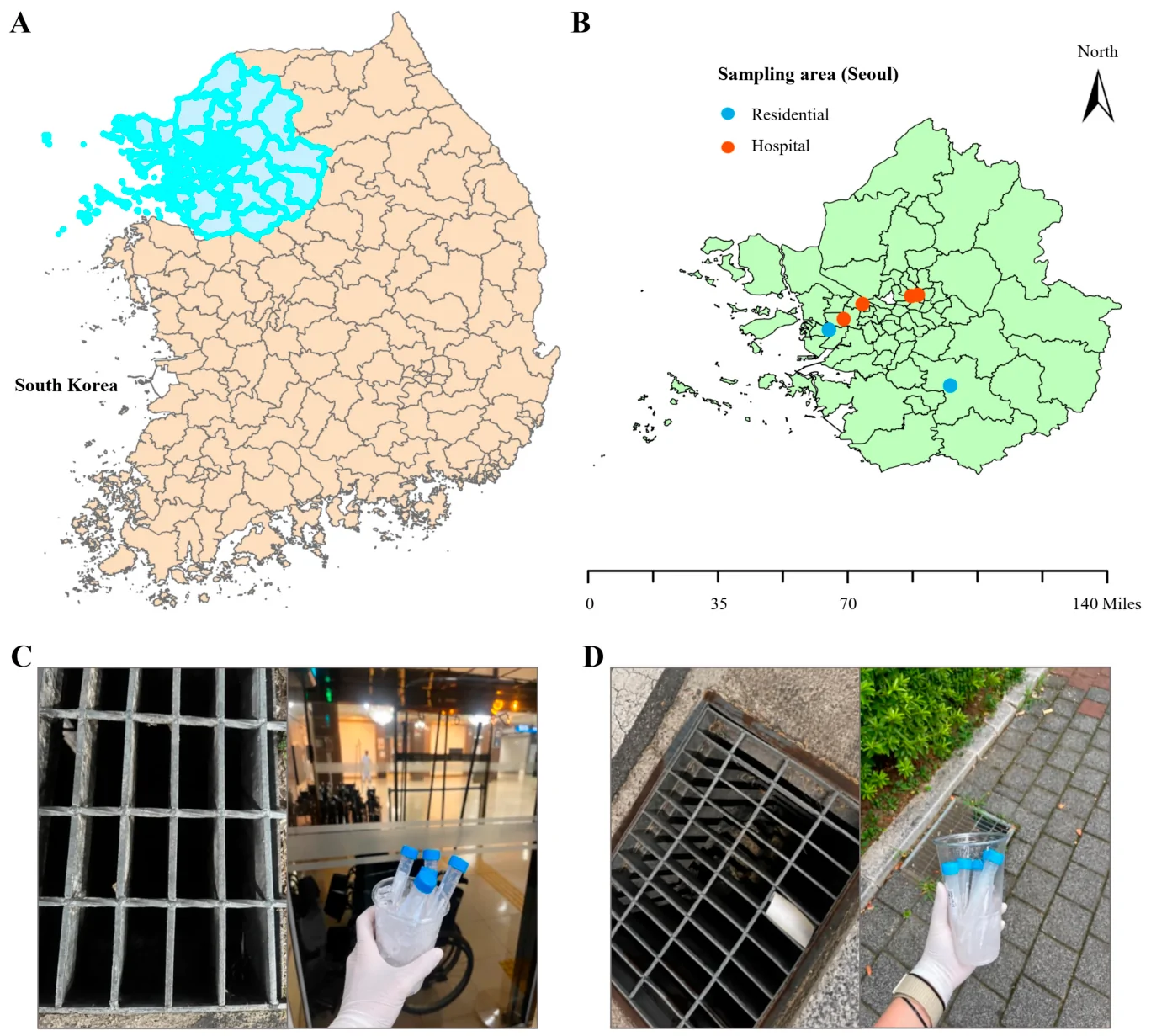
**Geographic distribution and environmental drain-sampling sites.** We collected environmental drain samples from hospital and residential locations across Seoul, Republic of Korea, for comparative microbial community analysis. (**A**) Map of South Korea highlighting Seoul as the geographic region selected for environmental drain sampling. (**B**) Detailed map of Seoul showing the spatial distribution of hospital and residential sampling locations, indicated by blue and orange markers. (**C**) Representative images of hospital drain environments and collection of drain samples using sterile sampling materials. (**D**) Representative images of residential drain environments and sample collection procedures. We used sterile sampling materials throughout the collection process to minimize potential contamination.

We collected environmental drain samples across Seoul, Republic of Korea, with hospital drains as the primary sampling environment and residential drains as a non-clinical comparison (Figure 1). Sampling included four hospital-associated and two residential locations, with representative sampling environments shown in Figure 1A–D. A total of 30 samples were collected, comprising 20 hospital drain samples and 10 residential drain samples, with five samples obtained from each location.

**Figure 2:**
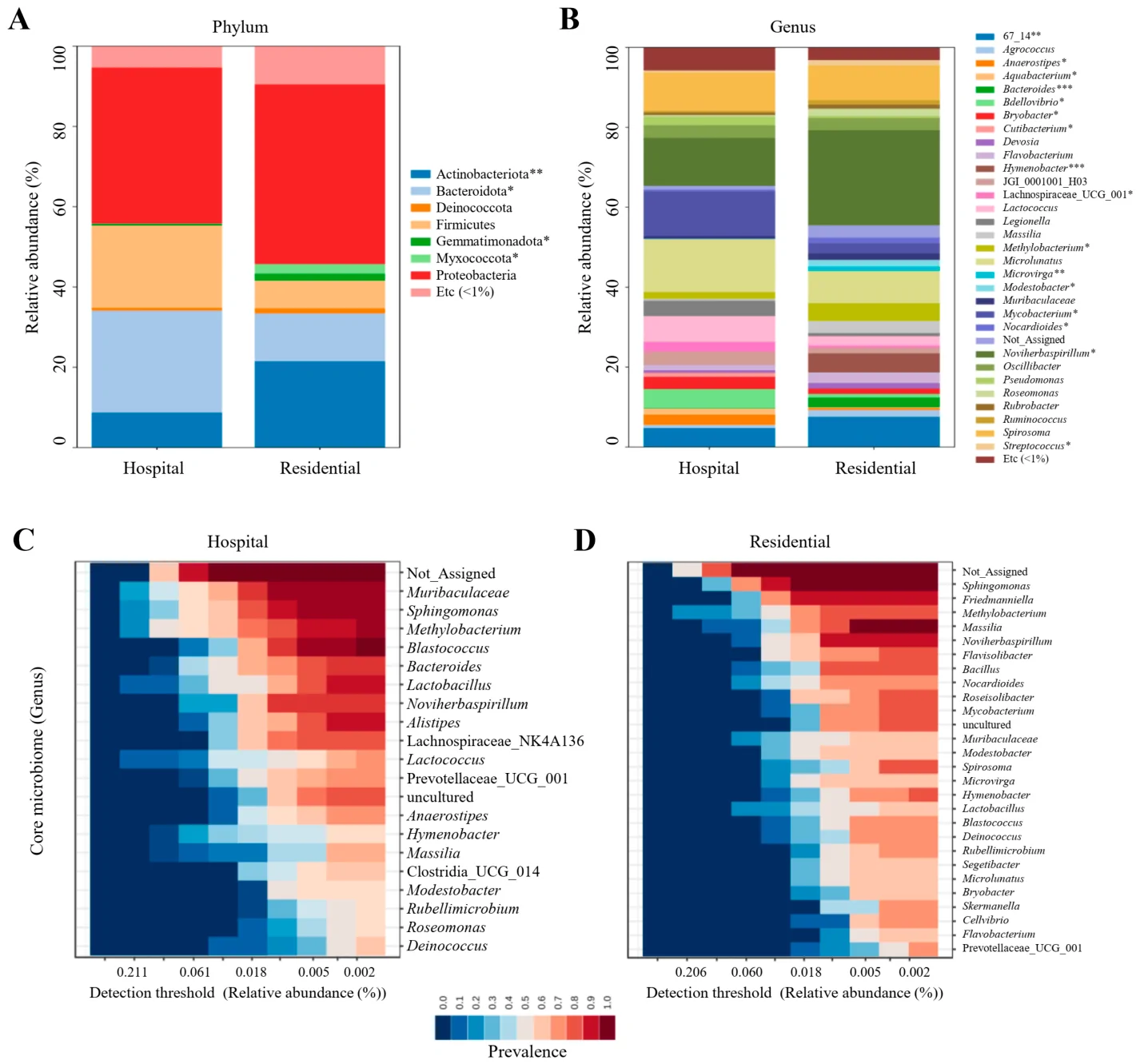
**Comparative taxonomic composition of hospital and residential drain microbiomes.** We compared microbial community composition between hospital and residential drain samples at the phylum and genus levels, along with prevalence patterns of the core microbiome. (**A**) Relative abundance of major bacterial phyla in hospital and residential samples. *Proteobacteria* and *Bacteroidota* were major components in both environments, while residential samples showed a higher relative abundance of *Actinobacteriota* and several low-abundance phyla. (**B**) Genus-level relative abundance profiles revealed distinct microbial compositions between hospital and residential samples, with differences in the distribution of dominant and low-abundance genera. (**C**,**D**) Prevalence heatmaps of core genera in hospital and residential samples across different relative-abundance detection thresholds. Statistical differences between groups were assessed using the Wilcoxon rank-sum test; *p* < 0.05 (*), *p* < 0.01 (**), and *p* < 0.001 (***).

Taxonomic profiling revealed distinct microbial compositions between hospital drains and the residential comparison across multiple taxonomic levels, including phylum, class, order, family, and genus (Figure 2; Table S1). At the phylum level, *Proteobacteria* were predominant in hospital drains (38.9%), with a comparable abundance observed in residential drains (44.8%) (Figure 2A). Hospital samples had substantially higher proportions of *Bacteroidota* and *Firmicutes*, whereas *Actinobacteriota* was more abundant in residential samples. At the genus level, hospital drains showed greater relative abundances of *Microlunatus*, *Mycobacterium*, *Lactococcus*, *Legionella*, *Anaerostipes*, *Pseudomonas*, and *Aquabacterium* (Figure 2B). In contrast, residential drains showed greater representation of *Noviherbaspirillum, Hymenobacter*, *Methylobacterium*, *Massilia*, *Flavobacterium*, *Bacteroides*, and *Microvirga*. *Spirosoma* remained abundant in both environments, representing 9.6% of hospital samples compared with 8.7% of residential samples. Core microbiome analysis further revealed distinct prevalence patterns within the two drainage environments. The analysis evaluated the prevalence of bacterial genera across samples at different detection thresholds based on their relative abundance, providing an overview of consistently detected microbial taxa within each environment. In these heatmaps, the horizontal axis represents the detection threshold (relative abundance, %), while the color gradient indicates the prevalence of each taxon across samples, with higher values representing detection in a greater proportion of samples. The hospital core included *Muribaculaceae*, *Sphingomonas*, *Methylobacterium*, *Blastococcus*, *Bacteroides*, *Lactobacillus*, *Alistipes*, and *Anaerostipes* across different relative-abundance thresholds (Figure 2C). In contrast, the residential core included *Sphingomonas*, *Methylobacterium*, *Mucilaginibacter*, *Flavobacterium*, *Bacillus*, *Roseomonas*, *Mycobacterium*, *Microbacterium*, and *Deinococcus* (Figure 2D). Collectively, the taxonomic composition plus core microbiome profiles identify a distinct hospital-associated microbial signature, characterized particularly by greater representation of *Mycobacterium*, *Lactococcus*, *Bdellovibrio*, *Legionella*, and *Pseudomonas.* At the same time, residential drains provide a contrasting non-clinical microbial reference.

### 2.2. Patterns of Alpha and Beta Microbial Diversity Across Drain Environments

**Figure 3:**
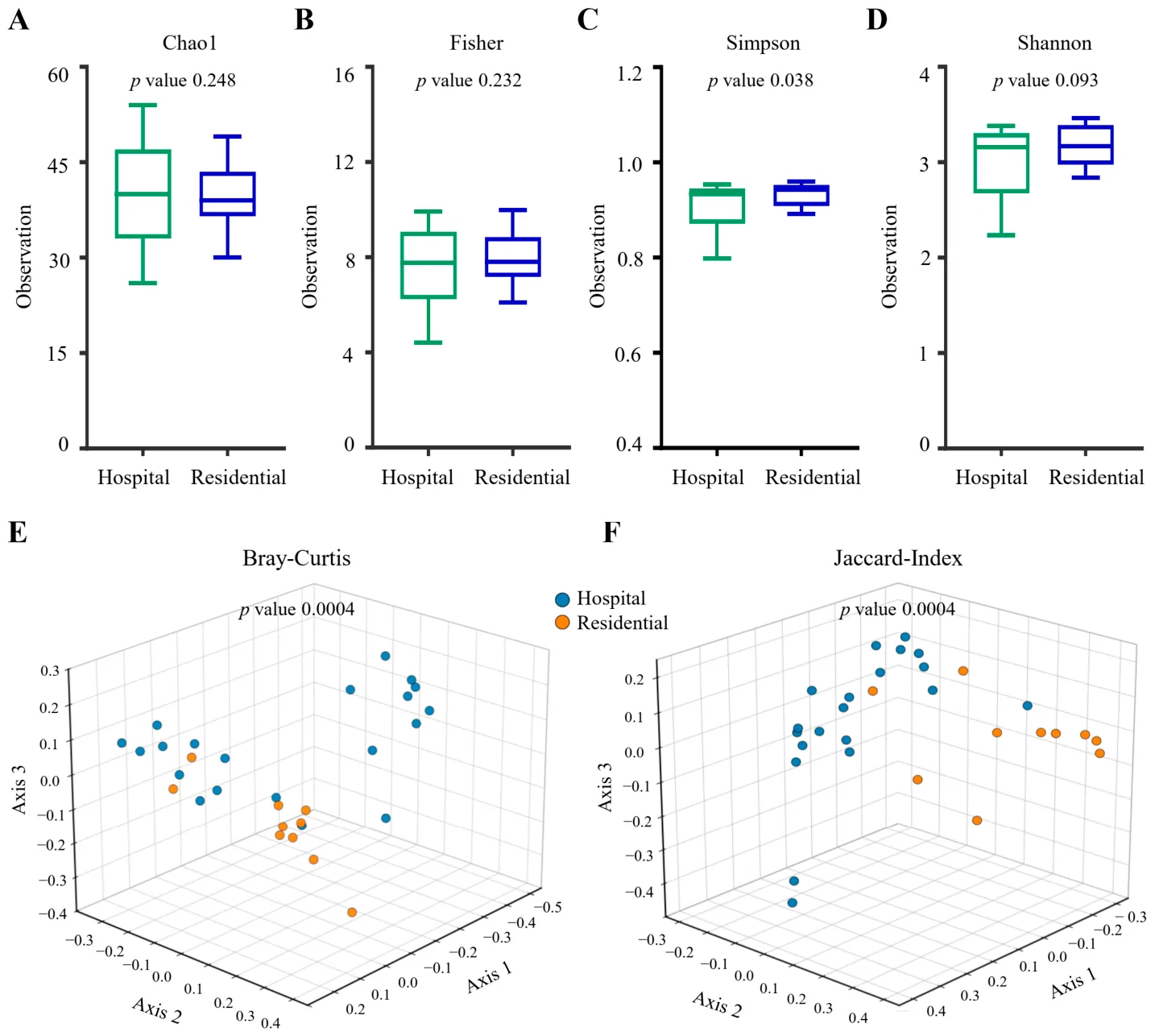
**Diversity analysis of microbial communities in hospital and residential drains.** We compared microbial diversity between hospital and residential drain samples using multiple alpha diversity indices and three-dimensional beta diversity analyses. (**A**–**D**) We assessed alpha diversity using the Chao1, Fisher, Simpson, and Shannon indices. Chao1 and Fisher indices showed no significant differences between the two environments. The Simpson index was significantly higher in residential samples, whereas the Shannon index showed a higher trend in residential samples but did not reach statistical significance. We assessed statistical significance for alpha diversity using an unpaired t-test. (**E**,**F**) Beta diversity analysis based on Bray–Curtis and Jaccard distances demonstrated distinct clustering patterns between hospital and residential microbial communities. Both Bray–Curtis and Jaccard analyses showed significant differences in overall community structure. We evaluated differences in overall community composition using PERMANOVA.

Microbial diversity analysis revealed clear differences in community structure between hospital and residential drains (Figure 3). Alpha-diversity analysis showed that overall species richness was relatively comparable between the two environments, as neither the Chao1 (*p* = 0.248) nor Fisher (*p* = 0.232) index differed significantly between hospital and residential samples (Figure 3A,B). In contrast, the Simpson index was significantly higher in residential samples than in hospital samples (*p* = 0.038), indicating greater community evenness in the residential environment (Figure 3C). The Shannon index also tended to be higher in residential samples, although the difference did not reach statistical significance (*p* = 0.093) (Figure 3D). These findings show that the main difference between the environments was more strongly associated with the distribution and evenness of bacterial taxa than with overall richness. Consistent with this observation, beta diversity analysis demonstrated pronounced differences in overall microbial community structure between the two environments. Three-dimensional ordination based on Bray–Curtis dissimilarity showed distinct spatial distributions of hospital and residential samples, with PERMANOVA confirming a highly significant difference in community composition (*p* = 0.0004) (Figure 3E). Similarly, Jaccard-based analysis, which emphasizes differences in taxon presence and absence, demonstrated significant separation between the two environments (*p* = 0.0004) (Figure 3F). Together, hospital and residential environments supported broadly comparable levels of microbial richness but harbored significantly different community configurations.

### 2.3. Community-Level Relationships Revealed by Hierarchical Clustering

**Figure 4:**
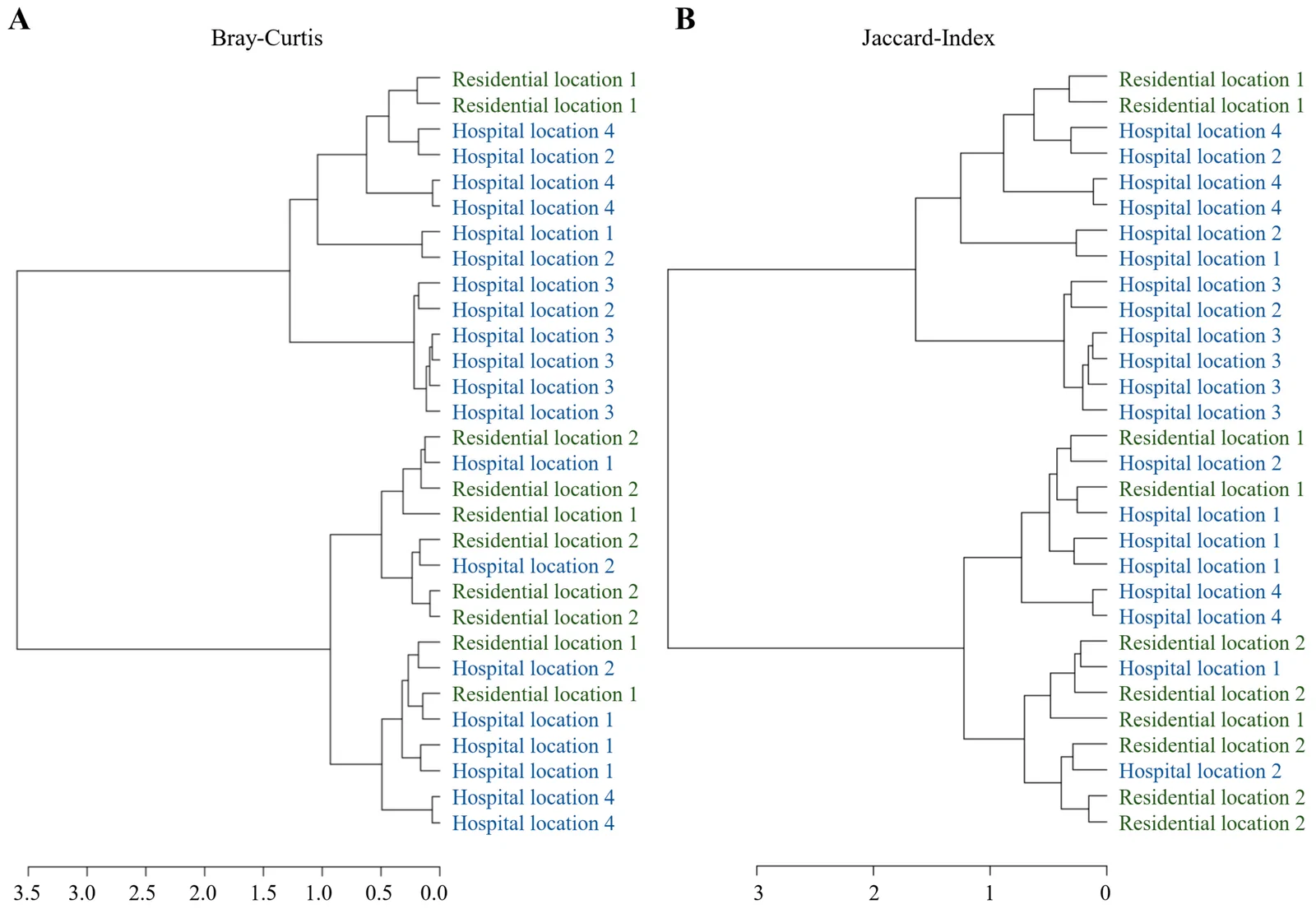
**Hierarchical clustering of hospital and residential drain microbial communities.** Hierarchical clustering was performed to assess similarities in microbial community composition among individual hospital and residential drain samples. Dendrograms were generated using (**A**) Bray–Curtis and (**B**) Jaccard distance metrics, representing abundance-based and presence/absence-based community dissimilarities, respectively. Samples grouped within the same or closely related branches exhibited greater microbial similarity, whereas longer branch distances reflected greater community dissimilarity. Both analyses showed partial environment-specific clustering, although several hospital and residential samples clustered together.

Hierarchical clustering further examined patterns of microbial community similarity among individual samples collected from hospital and residential environments (Figure 4). Samples were identified according to their respective sampling locations to facilitate the assessment of within-location similarities and between-environment differences. Bray–Curtis-based clustering, which considers differences in taxon abundance, separated the samples into several major branches, with groups of hospital and house samples forming relatively distinct clusters (Figure 4A). However, several samples from the two environments were positioned within closely related branches, indicating that some communities shared similar abundance profiles despite originating from different environments. A comparable pattern emerged with the Jaccard index, which reflects differences in taxon presence and absence (Figure 4B). Although hospital samples tended to cluster together in several branches and multiple house samples formed separate groups, samples from the two environments also intermixed. The broadly similar clustering patterns obtained with both distance metrics suggest that differences between hospital and residential microbiomes involve both the relative abundance and occurrence of bacterial taxa. Hierarchical clustering supports environment-associated structuring of the microbial communities while also showing microbial similarity and overlap between hospital and residential environments.

### 2.4. Taxonomic Signatures Associated with Drain Environments

**Figure 5:**
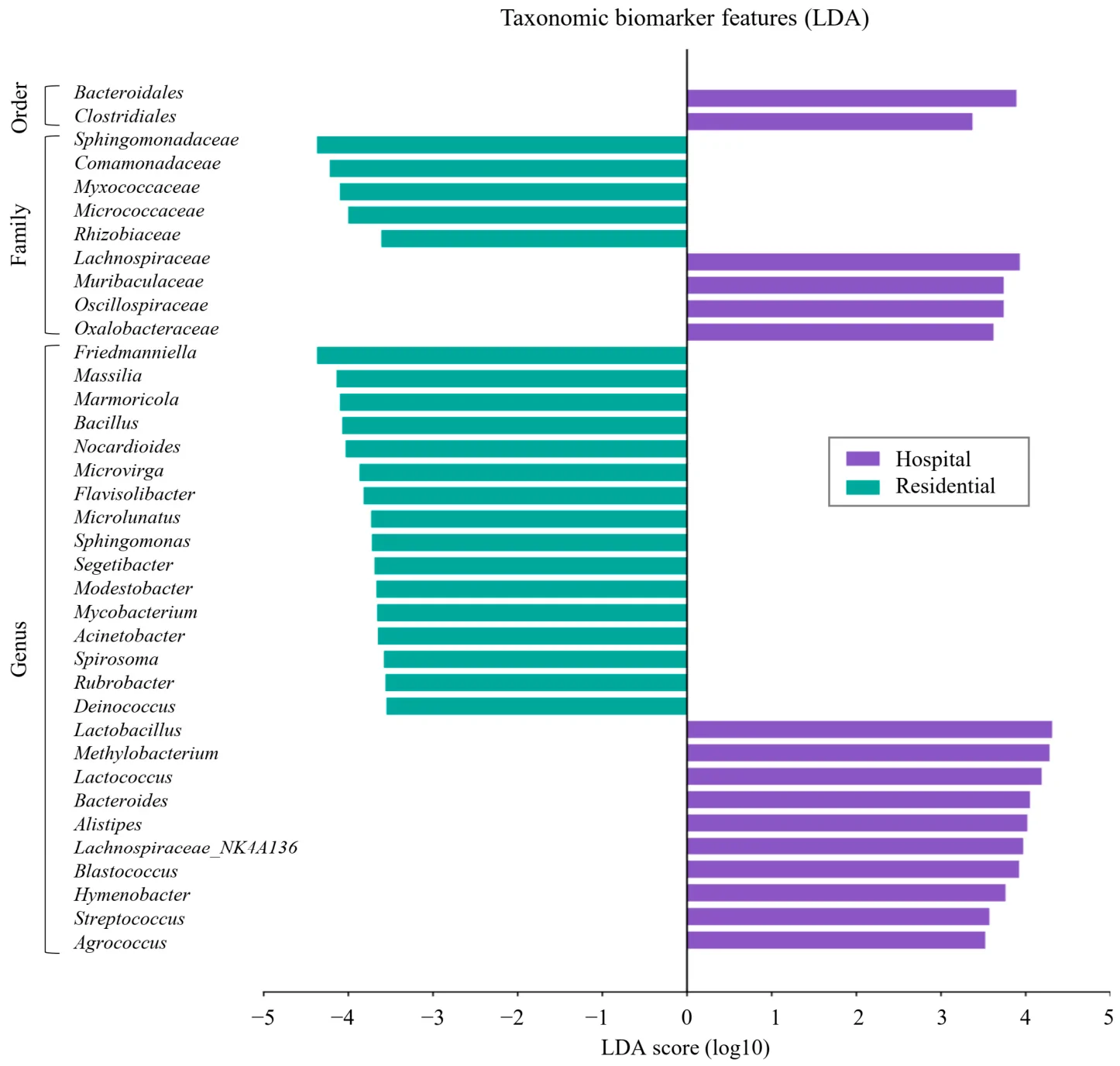
**Differential taxonomic biomarkers of hospital and residential drain microbiomes identified by LEfSe analysis.** Linear discriminant analysis (LDA) identified bacterial taxa that discriminated between hospital and residential drain microbial communities. The LDA score plot displays differentially enriched taxa at the order, family, and genus levels, with positive LDA scores indicating enrichment in hospital samples and negative scores indicating enrichment in residential samples. At the order level, *Bacteroidales* and *Clostridiales* were enriched in hospital samples. At the family level, *Lachnospiraceae*, *Muribaculaceae*, *Oscillospiraceae*, and *Oxalobacteraceae* were associated with hospital samples, whereas *Sphingomonadaceae*, *Comamonadaceae*, *Myxococcaceae*, *Micrococcaceae*, and *Rhizobiaceae* were enriched in residential samples. Several genus-level biomarkers also distinguished the two environments.

LEfSe analysis identified distinct taxonomic biomarkers that differentiated the microbial communities of hospital and residential environments (Figure 5). At the order level, *Bacteroidales* and *Clostridiales* were enriched in hospital samples, indicating a greater contribution of these taxonomic groups to the hospital-associated microbiome. At the family level, hospital samples were characterized by enrichment of *Lachnospiraceae*, *Muribaculaceae*, *Oscillospiraceae*, and *Oxalobacteraceae*, whereas residential samples were enriched in *Sphingomonadaceae*, *Comamonadaceae*, *Myxococcaceae*, *Micrococcaceae*, and *Rhizobiaceae*. More extensive differentiation was observed at the genus level. Hospital-associated biomarkers included *Lactobacillus*, *Methylobacterium*, *Lactococcus*, *Bacteroides*, *Alistipes*, *Blastococcus*, *Hymenobacter*, *Streptococcus*, and *Agrococcus*. In contrast, house samples were characterized by a broader range of discriminative genera, including *Friedmanniella*, *Massilia*, *Marmoricola*, *Bacillus*, *Nocardioides*, *Microvirga*, *Flavisolibacter*, *Microlunatus*, *Sphingomonas*, *Segetibacter*, *Modestobacter*, *Mycobacterium*, *Acinetobacter*, *Spirosoma*, *Rubrobacter*, and *Deinococcus*. The magnitude of the LDA scores further indicated strong discriminatory contributions of several taxa, with *Lactobacillus*, *Methylobacterium*, and *Lactococcus* among the prominent hospital-associated biomarkers. In contrast, *Friedmanniella*, *Massilia*, and several residential-associated families showed strong negative LDA scores. The LEfSe analysis revealed environment-specific taxonomic signatures across multiple phylogenetic levels and showed a broader spectrum of discriminative bacterial taxa in the residential environment than in the hospital environment.

### 2.5. Functional Divergence of Microbial Metabolic Pathways Between Hospital and Residential Drains

Functional pathway analysis revealed marked differences in the predicted metabolic potential of microbial communities between hospital and residential environments (Table 1). The analysis of predicted functional pathways identified the top 25 significantly differentiated pathways between the hospital and residential groups, with the majority (19/25, 76%) enriched in the hospital group and the remainder (6/25, 24%) enriched in the residential group. To facilitate biological interpretation, these pathways were classified into five major functional categories: amino acid biosynthesis and metabolism; nucleotide biosynthesis and salvage; cofactor, vitamin, and siderophore biosynthesis; cell envelope biosynthesis; and energy, fermentation, and other catabolic metabolism. Hospital-enriched pathways were dominated by core biosynthetic and anabolic functions, including aromatic amino acid biosynthesis (ARO-PWY, COMPLETE-ARO-PWY), folate biosynthesis (FOLSYN-PWY), peptidoglycan/cell-wall biosynthesis (PEPTIDOGLYCANSYN-PWY), nucleotide and cofactor biosynthesis (PWY-6121, PWY-6122, PWY-6277, PWY-6123, PWY-7208, PYRIDNUCSAL-PWY), and fermentative energy metabolism (FERMENTATION-PWY), with PWY-5104 showing the strongest discriminatory power overall (LDA = 0.892). In contrast, residential-enriched pathways centered on siderophore-mediated iron acquisition (ENTBACSYN-PWY, ALL-CHORISMATE-PWY) and several catabolic/salvage routes (PWY-7527, PWY-5022, P562-PWY, PWY-5304, PWY-5392), with PWY-7527 being the single most statistically significant pathway in the dataset. These findings suggest that the hospital-associated community is functionally shifted toward greater biosynthetic self-sufficiency. In contrast, the residential-associated community relies more heavily on iron-scavenging and catabolic strategies, pointing to distinct metabolic adaptations between the two environments. The predominance of hospital-enriched pathways among the significant biomarkers shows that taxonomic differences between hospital and residential microbial communities were accompanied by substantial shifts in their predicted functional profiles, particularly in pathways related to microbial biosynthesis, fermentation, and cellular metabolic processes.

## 3. Discussion

The microbial ecology of drainage systems is shaped by water flow, nutrient availability, human-derived inputs, cleaning practices, and the physicochemical characteristics of the surrounding environment [8]. Hospital drainage systems represent an important interface between healthcare facilities, urban wastewater infrastructure, and the broader aquatic environment [9]. Unlike conventional built-environment surfaces, hospital drains continuously receive water, along with microorganisms and organic materials, from intensive human activity and healthcare-associated processes [10]. Consequently, microbial communities in hospital drainage systems may provide information relevant not only to the hospital environment itself but also to downstream wastewater surveillance. This consideration is particularly relevant in densely urbanized regions such as Seoul, where wastewater is ultimately connected to receiving aquatic systems associated with the Han River watershed. In the present study, residential drains were therefore used primarily as a non-clinical environmental comparator to determine whether hospital drains carried a distinguishable microbial signature.

The taxonomic composition of the hospital drains provided clear evidence of an environment-associated microbial profile, with *Proteobacteria* representing the dominant phylum, followed by *Bacteroidota* and *Firmicutes*. *Bacteroidota* and *Firmicutes* were substantially more abundant in hospital than residential drains, whereas *Actinobacteriota* was considerably higher in residential samples. This broad phylum-level pattern is consistent with previous hospital wastewater studies reporting *Proteobacteria*, *Bacteroidota*, *Firmicutes*, and *Actinobacteriota* as major community members. However, their relative proportions vary according to hospital characteristics, geographic location, wastewater treatment, and sampling point [11,12]. At the genus level, hospital drains showed greater relative abundances of *Mycobacterium*, *Legionella*, *Pseudomonas*, *Lactococcus*, *Bdellovibrio*, *Anaerostipes*, and *Aquabacterium*. The greater abundance of *Pseudomonas*, *Legionella*, and *Mycobacterium* is particularly relevant to environmental surveillance because members of these genera are frequently detected in aquatic environments, plumbing systems, and biofilms, and previous hospital wastewater studies have similarly reported *Pseudomonas* and other opportunistic genera [13]. However, 16S rRNA amplicon sequencing cannot establish species identity, viability, virulence, or pathogenicity; therefore, these taxa should be considered environmental surveillance signals rather than evidence of direct health risk. Core microbiome analysis identified repeatedly detected hospital-associated taxa, including *Sphingomonas*, *Methylobacterium*, *Bacteroides*, *Lactobacillus*, *Alistipes*, and *Anaerostipes*, suggesting that the observed microbial profile was not driven solely by occasional microbial deposition. This aligns with previous studies showing the persistence and recolonization of hospital drain biofilms and supports the possibility that hospital drains can function as relatively stable microbial reservoirs that continuously contribute microorganisms to downstream wastewater streams [14,15].

Beyond these compositional differences, the alpha- and beta diversity findings further demonstrated that hospital drains supported a distinct microbial community structure compared with residential drains. Chao1 and Fisher richness did not differ significantly between the groups, whereas Simpson diversity was significantly lower in hospital drains, and Shannon diversity showed a similar trend without reaching statistical significance. These findings suggest that the main within-sample difference was community evenness rather than the overall number of detectable taxa, with hospital drains showing greater dominance by particular bacterial groups [16]. This pattern may reflect stronger environmental filtering within hospital drainage systems, potentially linked to differences in disinfectant exposure, antimicrobial residues, clinical wastewater inputs, nutrient availability, and water-use patterns. Consistent with this interpretation, previous studies have shown that anthropogenic pressures and disinfectant exposure can restructure built-environment microbial communities and select for tolerant populations [17]. Beta diversity showed more pronounced differentiation, with both Bray–Curtis and Jaccard analyses showing significant separation between hospital and residential samples. Similar source-dependent differences have been reported among hospital and domestic wastewater communities and across different stages of hospital wastewater treatment [18]. Importantly, the agreement between Bray–Curtis and Jaccard analyses indicates that the hospital-associated signature involved differences in both the relative abundance and occurrence of bacterial taxa rather than changes in only a few dominant organisms. These diversity patterns indicate that hospital drainage represents a distinct microbial environment and support the potential application of community-level microbial signatures for source-specific wastewater surveillance within the Seoul urban watershed [19].

Hierarchical clustering provided a complementary perspective by showing both environmental separation and partial overlap. Hospital samples frequently grouped within related branches under Bray–Curtis and Jaccard clustering, although several residential and hospital samples were interspersed. The absence of complete separation suggests that microbial community composition may be influenced not only by the hospital or residential environment but also by site-specific conditions and local environmental variability [20]. Samples collected from the same location may share similar microbial communities because of their exposure to comparable drainage conditions, potentially contributing to the observed clustering patterns. Such overlap is expected because both environments are components of urban drainage infrastructure and share fundamental ecological conditions, including moist surfaces, intermittent nutrient inputs, human-derived microorganisms, plumbing biofilms, and water-associated bacteria [21]. Previous work examining clinical wastewater from sewage systems through receiving environments similarly found that wastewater sources have characteristic bacterial communities, but environmental processing alters these signatures along the wastewater pathway [22,23]. Therefore, the partial overlap observed here should not be interpreted as contradicting the significant PERMANOVA results. Rather, it suggests a common urban drain microbiome overlaid by hospital-specific ecological selection [24].

To further resolve the taxa underlying these differences, LEfSe analysis further identified specific taxa contributing to the hospital-associated microbial signature. At the order level, *Bacteroidales* and *Clostridiales* were enriched in hospital drains, while the hospital-associated families included *Lachnospiraceae*, *Muribaculaceae*, *Oscillospiraceae*, and *Oxalobacteraceae*. At the genus level, the analysis identified several hospital-associated discriminative taxa, including *Lactobacillus*, *Methylobacterium*, *Lactococcus*, *Bacteroides*, *Alistipes*, *Blastococcus*, *Hymenobacter*, *Streptococcus*, and *Agrococcus*. The enrichment of *Bacteroidales* and several gastrointestinal-associated bacterial groups may be particularly informative because hospital wastewater receives substantial human-derived microbial inputs. Previous hospital wastewater studies have likewise reported complex communities containing human-associated and environmental organisms, including Bacteroides and other fecal-associated taxa [25]. A recent profiling study of hospital wastewater identified *Proteobacteria, Firmicutes*, and *Bacteroidetes* as predominant phyla and highlighted *Bacteroides* among genera detected in hospital wastewater discharge [26]. These hospital-associated biomarkers could therefore be useful candidates for future source-tracking studies. However, LEfSe demonstrates statistical discrimination rather than environmental transport; their presence in hospital drains does not establish that these organisms survive wastewater treatment or reach the Han River.

The predicted functional profile provides additional evidence that the hospital drain microbiome represents an ecologically distinct microbial community. Remarkably, 19 of the 25 most significantly differentiated pathways were enriched in hospital samples, whereas only six were enriched in residential samples. Hospital-enriched functions included aromatic amino acid biosynthesis, folate biosynthesis, peptidoglycan biosynthesis, nucleotide/cofactor biosynthesis, and fermentation, with FERMENTATION-PWY reaching 25.96% in hospital samples compared with 17.77% in residential drains. This predominance of biosynthetic and central metabolic functions may indicate a community adapted to nutrient availability and repeated organic inputs within the hospital drainage environment [27]. Environmental metagenomic studies have shown that differences in microbial community composition can reflect habitat-specific metabolic adaptation, while wastewater communities are particularly influenced by substrate composition and physicochemical conditions [28]. The finding is therefore consistent with the taxonomic differentiation observed in the present study and suggests that hospital selection acts at both compositional and predicted functional levels.

Overall, the present findings establish hospital drains in Seoul as distinct microbial ecosystems relative to residential drains, characterized by differences in taxonomic composition, community evenness, overall community structure, discriminatory taxa, and predicted metabolic potential. The most important environmental implication is not that this study demonstrates contamination of the Han River, but that it identifies a potential upstream microbial source that warrants direct source-to-receptor investigation. Evidence from other wastewater systems demonstrates that hospital-associated microorganisms, antimicrobial residues, and resistance determinants can persist through wastewater treatment and influence receiving rivers. Future studies should therefore extend sampling from hospital drainage outlets through municipal wastewater infrastructure to treatment-plant influent and effluent and finally to upstream and downstream Han River locations. Combining 16S profiling with shotgun metagenomics, quantitative ARG analysis, cultivation of priority organisms, water-quality measurements, and longitudinal sampling would allow direct assessment of whether the hospital-associated signatures identified here contribute measurably to the Han River’s microbial ecology and public health burden.

## 4. Methods

### 4.1. Environmental Sample Collection

We collected environmental drain samples from hospital and residential locations in Seoul, Gyeonggi-do, and Incheon, Republic of Korea, between July and August 2026. A total of 30 samples were collected, comprising 20 hospital-associated drain samples from four sampling locations and 10 residential drain samples from two sampling locations, with five samples obtained from each location. Hospital-associated samples were collected from drainage areas near Korea University Hospital (Hospital location 1), Inje University Sanggye Paik Hospital (Hospital location 2), Soonchunhyang University Bucheon Hospital (Hospital location 3), and Kyung Hee University Medical Center (Hospital location 4). Residential drain samples were obtained from drainage areas in Yongin, Gyeonggi-do (House location 1), and Incheon (House location 2). Environmental drain samples were collected by swabbing accessible surfaces of roadside drainage channels to recover surface-associated microbial material using sterile swabs. Following collection, the swabs were placed in sterile collection tubes and transported to the laboratory under refrigerated conditions (approximately 4 °C). Upon arrival, the samples were stored at −80 °C until further processing.

### 4.2. Genomic DNA Extraction

We collected samples from hospital and residential drains and stored them at −80 °C for long-term storage. We extracted genomic DNA using the DNeasy PowerSoil Pro Kit (Qiagen, Hilden, Germany) following the manufacturer’s standard protocol [29]. We measured DNA concentration on a Qubit 4 Fluorometer (Thermo Fisher Scientific, Waltham, MA, USA) and verified DNA integrity by 0.8% agarose gel electrophoresis. We stored purified DNA at −20 °C until further use. We rechecked concentration, purity, and integrity at each subsequent step during extraction, library preparation, and before sequencing to ensure consistency across samples. We carried out all extraction and library work under aseptic conditions and ran no-template controls alongside experimental samples to monitor for contamination.

### 4.3. 16S rRNA Gene Amplification and Library Construction

We amplified the V4 hypervariable region of the bacterial 16S rRNA gene by PCR using primers described in a previous study [30]. We carried out amplification on a Veriti 96-well Thermal Cycler (Applied Biosystems, Foster City, CA, USA) following Illumina’s standard amplicon PCR protocol, including a no-template negative control (blank extraction and PCR blank) in each run to rule out reagent or cross-contamination. Each 25 μL reaction contained 10 ng of template DNA, 5 μM each of the forward and reverse primers, KAPA ReadyMix (Kapa Biosystems, Wilmington, MA, USA), and nuclease-free water. We cleaned PCR products with AMPure XP magnetic beads (Beckman Coulter, Brea, CA, USA) to remove primer dimers and residual reagents. Sample-specific barcodes were then added by indexing PCR using the Nextera XT DNA Library Preparation Kit (Illumina, San Diego, CA, USA). After a second round of bead purification, we normalized individual libraries to 1 nM in 10 mM Tris buffer and pooled them in equal volumes (5 μL per sample). The pooled library (50 pmol) was spiked with 30% PhiX control (Illumina, San Diego, CA, USA) to increase base-call diversity and sequenced on the Illumina iSeq100 platform to generate paired-end reads for downstream bioinformatic analysis [31].

### 4.4. 16S rRNA Amplicon Bioinformatics

Raw FASTQ reads generated from the iSeq100 run were processed in QIIME 2 (version 2021.11). We performed read demultiplexing and quality-based trimming with the q2-demux plugin, and denoising, including chimera removal and error correction, with DADA2 to generate amplicon sequence variants (ASVs) [32]. We filtered features occurring fewer than five times across the dataset at the command-line stage. We aligned representative ASV sequences with MAFFT and then built a phylogenetic tree with FastTree2. We generated sequencing depth, quality-filtering statistics, and ASV summaries following the workflow described in our earlier work [33]. ASVs were clustered into operational taxonomic units (OTUs) at a 97% sequence-similarity threshold, and representative sequences from each OTU were taxonomically classified using the RDP Naïve Bayesian Classifier, referencing the Human Microbiome Database from phylum to genus level [34]. Sequences were additionally cross-referenced against the Greengenes database (v13-8) for taxonomic assignment; where multiple sequence clusters belonged to the same genus, Greengenes-specific suffixes (e.g., “_g23”) were retained to distinguish them [35]. We visualized community composition using the METAGENAssist web platform [36]. We generated rarefaction curves to assess sampling depth and bacterial diversity coverage [37]. Alpha diversity (within-sample richness and evenness) was estimated using the Chao1 and Fisher richness indices together with observed OTU counts, and diversity was further quantified using the Shannon and Simpson indices [38,39,40]. Beta diversity (between-sample dissimilarity) was calculated using Bray–Curtis and Jensen–Shannon divergence distance metrics, and group-level differences were tested for statistical significance using PERMANOVA [41,42]. We identified bacterial taxa that differentiated the study groups by combining the Kruskal–Wallis H test with linear discriminant analysis effect size (LEfSe) analysis (α = 0.05, LDA score cutoff = 2.0) [42]. We predicted functional microbiome profiles based on MetaCyc pathway annotations using the MicrobiomeAnalyst platform [43]. We used predicted pathway abundances for downstream analyses after filtering and normalization within the pipeline [30].

### 4.5. Statistical Analysis

We set statistical significance at *p* < 0.05 throughout. Alpha diversity was compared using the richness (Chao1 and Fisher) and diversity (Shannon and Simpson) indices described above, and beta diversity was evaluated by PERMANOVA on Bray–Curtis and Jensen–Shannon distance matrices [44]. Differences in taxonomic abundance at the phylum-to-genus level between the hospital and house groups were assessed using the Wilcoxon rank-sum test, with significance denoted as * (*p* < 0.05), ** (*p* < 0.01) and *** (*p* < 0.001). Microbial biomarker discovery combined the non-parametric Kruskal–Wallis H test with LEfSe, which incorporates effect-size estimation to control for multiple comparisons [45].

## 5. Conclusions

**Figure 6:**
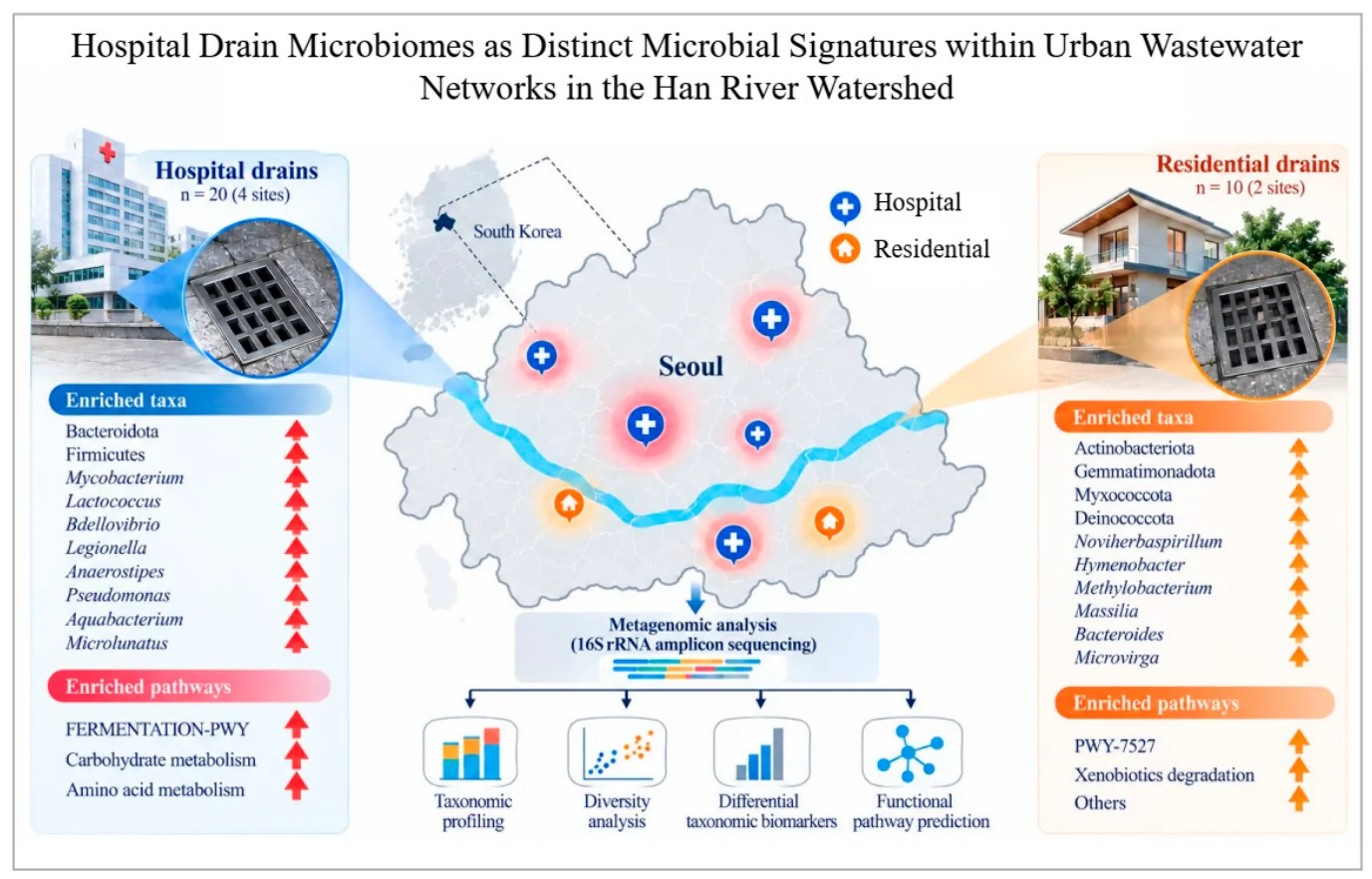
**Graphical abstract of hospital drain microbiomes as distinct microbial signatures within urban wastewater networks.** Hospital and residential drain samples collected across Seoul, Republic of Korea, were characterized using 16S rRNA amplicon sequencing. Comparative analyses revealed distinct microbial profiles between the two environments, encompassing taxonomic composition, microbial diversity, differential taxonomic biomarkers, and predicted functional pathways. The characteristic microbial and functional signatures observed in hospital drains highlight their distinct ecological profile within the urban wastewater network and provide a foundation for understanding how healthcare-associated microbial signatures may integrate into municipal wastewater systems and the wider Han River watershed.

This study identifies hospital drains in Seoul as distinct microbial ecosystems within the urban wastewater environment, with clear differences from residential drains in taxonomic composition, community diversity, microbial structure, discriminative taxa, and predicted functional pathways. Notably, the higher relative abundances of *Mycobacterium*, *Legionella*, and *Pseudomonas* in hospital drains highlight these genera as potential targets for future pathogen surveillance and public health risk assessment. The enrichment of biosynthetic pathways further indicates distinct predicted metabolic characteristics of hospital-associated microbial communities. These findings highlight healthcare drainage systems as potential upstream sources of microorganisms entering municipal wastewater networks and provide a baseline for evaluating their downstream environmental relevance within the Han River watershed. Future studies integrating species-level pathogen identification, antimicrobial-resistance profiling, and source-to-receptor monitoring will be essential to determine their environmental and public health significance. The graphical abstract provides an overview of the key findings and highlights the distinct microbial characteristics of hospital drains within the urban wastewater network (Figure 6).

## Figures and Tables

**Table 1:** Abundant functional pathways between hospital and residential drain microbiomes.

Functional Pathway	Hospital (%)	Residential (%)	LDA Score	*p*-Value	FDR	Enriched in
**A. Amino acid biosynthesis and metabolism**						
*PWY-7527*	0.095	**3.060**	**−0.395**	4.07 × 10^−6^	8.41 × 10^−4^	Residential
*PWY-5022*	8.477	**16.716**	**−0.709**	1.98 × 10^−5^	8.41 × 10^−4^	Residential
*ALL-CHORISMATE-PWY*	5.766	**12.759**	**−0.653**	2.92 × 10^−5^	8.41 × 10^−4^	Residential
*ARO-PWY*	**39.364**	33.380	**0.601**	4.29 × 10^−5^	8.41 × 10^−4^	Hospital
*COMPLETE-ARO-PWY*	**41.447**	35.110	**0.620**	5.18 × 10^−5^	8.41 × 10^−4^	Hospital
*PWY-6163*	**38.592**	32.179	**0.624**	6.24 × 10^−5^	8.41 × 10^−4^	Hospital
*PWY-5104*	**31.614**	18.010	**0.892**	7.51 × 10^−5^	8.41 × 10^−4^	Hospital
**B. Nucleotide biosynthesis and salvage**						
*PWY-5686*	**45.913**	36.299	**0.764**	5.18 × 10^−5^	8.41 × 10^−4^	Hospital
*PWY-6121*	**46.444**	36.619	**0.772**	6.24 × 10^−5^	8.41 × 10^−4^	Hospital
*PWY-6122*	**46.227**	36.630	**0.763**	6.24 × 10^−5^	8.41 × 10^−4^	Hospital
*PWY-6277*	**46.227**	36.630	**0.763**	6.24 × 10^−5^	8.41 × 10^−4^	Hospital
*PWY-6123*	**39.989**	31.061	**0.738**	6.24 × 10^−5^	8.41 × 10^−4^	Hospital
*PWY-7208*	**46.650**	38.112	**0.722**	7.51 × 10^−5^	8.41 × 10^−4^	Hospital
**C. Cofactor, vitamin and siderophore biosynthesis**						
*ENTBACSYN-PWY*	2.244	**7.726**	**−0.573**	4.29 × 10^−5^	8.41 × 10^−4^	Residential
*FOLSYN-PWY*	**36.556**	29.598	**0.651**	6.24 × 10^−5^	8.41 × 10^−4^	Hospital
*PWY-6612*	**34.776**	28.035	**0.641**	6.24 × 10^−5^	8.41 × 10^−4^	Hospital
*PYRIDNUCSAL-PWY*	**29.756**	24.224	**0.576**	6.24 × 10^−5^	8.41 × 10^−4^	Hospital
*PWY-6147*	**39.187**	30.772	**0.717**	7.51 × 10^−5^	8.41 × 10^−4^	Hospital
**D. Cell envelope biosynthesis**						
*PWY-7315*	**7.189**	2.266	**0.539**	4.29 × 10^−5^	8.41 × 10^−4^	Hospital
*PWY-6385*	**39.766**	31.360	**0.716**	7.51 × 10^−5^	8.41 × 10^−4^	Hospital
*PEPTIDOGLYCANSYN-PWY*	**40.146**	31.932	**0.708**	7.51 × 10^−5^	8.41 × 10^−4^	Hospital
**E. Energy, fermentation and other catabolic metabolism**						
*FERMENTATION-PWY*	**25.960**	17.769	**0.707**	1.62 × 10^−5^	8.41 × 10^−4^	Hospital
*PWY-5392*	0.115	**1.720**	**−0.256**	3.62 × 10^−5^	8.41 × 10^−4^	Residential
*P562-PWY*	2.275	**6.852**	**−0.517**	6.24 × 10^−5^	8.41 × 10^−4^	Residential
*PWY-5304*	1.693	**5.959**	**−0.496**	6.24 × 10^−5^	8.41 × 10^−4^	Residential

Pathways (MetaCyc) are grouped into functional categories and ranked by ascending *p*-value within each category (top 25 of the significant biomarkers; all FDR < 0.001). Relative abundance is expressed as mean percentage; the higher value for each pathway is shown in bold. Positive LDA scores indicate enrichment in hospital drains and negative scores indicate enrichment in residential drains. FDR, false discovery rate; LDA, linear discriminant analysis.

## Data Availability

The sequencing data are associated with the NCBI BioProject database (BioProject ID: PRJNA1540402, accessible at https://www.ncbi.nlm.nih.gov/bioproject/1540402).

## Supplementary Materials

The following supporting information can be downloaded at https://www.mdpi.com/article/10.3390/antibiotics15101041/s1: Table S1: Taxonomic profile of the samples from hospital and residential groups.
