# Supplementary material for: Hospital Drain Microbiomes as Distinct Microbial Signatures Within Urban Wastewater Networks in the Han River Watershed
Source: Antibiotics (Basel). 2026 Oct 21;15(10):1041. doi: 10.3390/antibiotics15101041 (PMC13700792; doi:10.3390/antibiotics15101041)
Supplement: Supplementary file 1 [file antibiotics-15-01041-s001.zip › antibiotics-4614754-supplementary.pdf]

Supplementary Information

Table S1. Taxonomic profile of the samples from hospital and residential groups.

Taxa		Hospital							Residential						
		Average	SD	Max	Min	1st Q	2nd Q	3rd Q	Average	SD	Max	Min	1st Q	2nd Q	3rd Q
Phylum	*Actinobacteriota*	8.7	6.3	25.1	1.3	5.0	7.3	11.5	21.5	5.1	30.3	10.5	20.0	21.3	23.3
	Bacteroidota	25.4	13.7	50.4	3.7	15.3	25.2	37.7	11.9	5.2	22.2	5.9	8.6	9.8	13.9
	Deinococcota	0.7	1.2	4.4	0.0	0.0	0.2	0.6	1.3	1.4	4.5	0.0	0.2	0.8	2.1
	Firmicutes	20.5	19.4	68.6	0.0	4.1	18.3	30.4	6.9	6.0	17.3	0.4	2.3	4.5	11.0
	Gemmatimonadota	0.4	0.7	1.9	0.0	0.0	0.0	0.3	1.7	1.4	4.1	0.0	0.5	1.9	2.6
	Myxococcota	0.1	0.2	0.9	0.0	0.0	0.0	0.0	2.4	4.0	11.6	0.0	0.0	0.5	2.0
	Proteobacteria	38.9	23.6	78.9	2.9	17.1	35.6	64.8	44.8	9.1	56.3	28.8	44.4	46.6	49.4
Class	*Actinobacteria*	8.7	6.3	25.7	1.4	4.9	7.5	10.7	21.5	4.4	26.9	11.1	20.6	22.0	23.6
	Alphaproteobacteria	29.2	21.2	74.6	1.5	10.7	25.3	42.6	31.1	10.7	49.4	15.8	23.3	29.1	36.8
	Bacilli	11.4	16.7	59.7	0.0	1.8	6.2	11.3	5.8	4.9	13.9	0.4	2.4	4.0	8.6
	Bacteroidia	26.5	14.2	52.3	4.0	16.0	26.1	39.8	12.7	5.5	23.5	6.3	9.5	10.6	14.7
	Clostridia	10.0	8.7	24.1	0.0	1.7	8.2	19.5	1.6	1.8	4.5	0.0	0.0	1.1	2.5
	Deinococci	0.7	1.2	4.5	0.0	0.0	0.2	0.6	1.4	1.5	4.8	0.0	0.2	0.9	2.2
	Gammaproteobacteria	11.6	7.8	29.7	1.5	5.8	9.1	16.4	17.2	11.1	40.4	7.1	8.9	12.9	24.4
	Gemmatimonadetes	0.4	0.7	2.0	0.0	0.0	0.0	0.4	1.8	1.4	4.3	0.0	0.6	2.0	2.8
	Myxococcia	0.1	0.2	1.0	0.0	0.0	0.0	0.0	2.6	4.4	12.5	0.0	0.0	0.6	2.2
	Rubrobacteria	0.4	0.7	2.6	0.0	0.0	0.0	0.5	1.1	2.8	9.0	0.0	0.0	0.0	0.8
Order	*Acetobacterales*	0.7	0.7	2.0	0.0	0.0	0.6	1.1	1.8	1.7	4.5	0.0	0.0	2.1	3.1
	Bacillales	0.1	0.4	1.8	0.0	0.0	0.0	0.0	2.4	2.5	7.8	0.0	1.1	1.4	4.0
	Bacteroidales	20.6	17.4	48.5	0.0	4.3	17.2	39.2	4.4	5.3	14.8	0.0	0.1	3.0	6.6
	Burkholderiales	8.9	7.0	27.5	0.9	5.1	6.7	11.5	13.4	8.6	32.8	6.0	7.7	10.2	14.1
	Chitinophagales	1.6	2.0	5.9	0.0	0.0	0.5	2.7	4.6	3.1	8.3	0.0	2.7	4.9	7.1
	Clostridia_UCG_014	1.0	1.1	3.0	0.0	0.0	0.6	1.8	0.0	0.0	0.0	0.0	0.0	0.0	0.0
	Corynebacteriales	0.5	0.7	2.7	0.0	0.0	0.0	0.8	1.4	1.2	4.1	0.0	0.6	1.3	1.9
	Cytophagales	4.0	5.4	17.4	0.0	0.1	1.0	7.4	3.1	2.5	8.0	0.3	0.9	3.1	4.2
	Deinococcales	0.7	1.2	4.5	0.0	0.0	0.2	0.6	1.4	1.5	4.8	0.0	0.2	0.9	2.2
	Enterobacterales	1.1	1.4	4.7	0.0	0.2	0.7	1.1	0.5	1.2	4.0	0.0	0.0	0.0	0.4
	Frankiales	3.8	2.5	11.7	0.8	2.3	3.5	4.7	3.9	2.6	7.4	0.0	1.8	4.0	5.7
	Gemmatimonadales	0.4	0.7	2.0	0.0	0.0	0.0	0.4	1.8	1.4	4.3	0.0	0.6	2.0	2.8
	Lachnospirales	6.5	5.3	15.4	0.0	1.5	5.9	12.0	1.3	1.3	3.4	0.0	0.0	1.1	2.2
	Lactobacillales	11.3	16.8	59.7	0.0	1.5	6.2	11.3	3.3	4.8	12.4	0.0	0.5	1.3	2.7
	Micrococcales	2.4	2.4	8.2	0.0	1.0	1.9	2.4	4.1	2.4	7.6	1.3	2.0	3.7	5.7
	Myxococcales	0.1	0.2	1.0	0.0	0.0	0.0	0.0	2.6	4.4	12.5	0.0	0.0	0.6	2.2

	Oscillospirales	2.4	2.4	6.6	0.0	0.0	1.7	4.8	0.3	0.5	1.1	0.0	0.0	0.0	0.8
	Propionibacteriales	2.0	2.4	8.9	0.0	0.6	1.4	1.9	12.1	3.6	19.6	7.6	9.7	12.0	13.8
	Pseudomonadales	1.0	2.2	9.1	0.0	0.0	0.1	0.7	2.1	4.5	14.7	0.0	0.1	0.6	1.0
	Rhizobiales	15.3	13.1	41.4	0.0	3.1	14.5	21.5	11.7	10.1	31.8	3.8	5.1	7.8	12.7
	Rhodobacterales	1.2	1.4	4.5	0.0	0.0	0.6	1.9	1.5	1.5	4.6	0.0	0.4	1.2	2.3
	Rubrobacterales	0.4	0.7	2.6	0.0	0.0	0.0	0.5	1.1	2.8	9.0	0.0	0.0	0.0	0.8
	Sphingomonadales	11.2	10.5	31.2	0.0	2.2	7.3	18.2	14.9	6.4	25.5	6.6	10.9	13.4	18.7
Family	*Acetobacteraceae*	0.7	0.7	2.0	0.0	0.0	0.6	1.1	1.8	1.7	4.5	0.0	0.0	2.1	3.1
	Bacillaceae	0.1	0.4	1.8	0.0	0.0	0.0	0.0	2.4	2.5	7.8	0.0	1.1	1.4	4.0
	Bacteroidaceae	4.8	4.3	12.4	0.0	0.7	3.5	9.8	0.9	1.2	3.1	0.0	0.0	0.5	1.3
	Beijerinckiaceae	13.6	13.2	38.6	0.0	1.9	12.0	20.8	10.1	10.5	31.8	1.1	4.3	6.1	9.1
	Chitinophagaceae	1.6	2.0	5.9	0.0	0.0	0.5	2.7	4.6	3.1	8.3	0.0	2.7	4.9	7.1
	Clostridia_UCG_014	1.0	1.1	3.0	0.0	0.0	0.6	1.8	0.0	0.0	0.0	0.0	0.0	0.0	0.0
	Comamonadaceae	2.7	2.3	8.0	0.0	0.8	2.3	3.7	5.4	4.0	13.0	0.0	3.1	4.6	5.9
	Deinococcaceae	0.7	1.2	4.5	0.0	0.0	0.2	0.6	1.4	1.5	4.8	0.0	0.2	0.9	2.2
	Enterobacteriaceae	1.1	1.4	4.7	0.0	0.2	0.7	1.1	0.5	1.2	4.0	0.0	0.0	0.0	0.4
	Gemmatimonadaceae	0.4	0.7	2.0	0.0	0.0	0.0	0.4	1.8	1.4	4.3	0.0	0.6	2.0	2.8
	Geodermatophilaceae	3.7	2.5	11.7	0.8	2.3	3.2	4.4	3.0	2.0	5.4	0.0	1.8	3.4	4.5
	Hymenobacteraceae	3.2	4.4	15.0	0.0	0.1	0.8	6.5	1.4	1.3	4.2	0.0	0.4	1.2	2.0
	Lachnospiraceae	6.5	5.3	15.4	0.0	1.5	5.9	12.0	1.3	1.3	3.4	0.0	0.0	1.1	2.2
	Lactobacillaceae	6.4	10.3	37.3	0.0	0.6	3.0	5.9	2.3	3.4	9.5	0.0	0.0	0.8	2.2
	Microbacteriaceae	1.5	1.6	5.9	0.0	0.0	1.4	1.9	1.2	1.6	5.1	0.0	0.0	0.6	1.7
	Micrococcaceae	0.7	1.6	6.9	0.0	0.0	0.0	0.5	2.2	2.0	4.6	0.0	0.4	2.1	4.1
	Moraxellaceae	0.8	2.0	8.2	0.0	0.0	0.0	0.6	1.7	4.6	14.7	0.0	0.0	0.2	0.6
	Muribaculaceae	11.1	9.2	25.8	0.0	2.2	10.0	20.3	2.5	3.1	8.3	0.0	0.0	1.4	4.4
	Mycobacteriaceae	0.5	0.7	2.7	0.0	0.0	0.0	0.8	1.4	1.2	4.1	0.0	0.6	1.3	1.9
	Myxococcaceae	0.1	0.2	1.0	0.0	0.0	0.0	0.0	2.6	4.4	12.5	0.0	0.0	0.6	2.2
	Nocardioidaceae	1.4	2.1	8.6	0.0	0.0	1.1	1.7	6.1	4.5	14.8	0.0	3.7	5.5	7.1
	Oscillospiraceae	2.0	2.0	5.4	0.0	0.0	1.6	3.5	0.3	0.5	1.1	0.0	0.0	0.0	0.6
	Oxalobacteraceae	6.2	6.0	23.9	0.5	2.2	4.2	7.0	8.0	5.1	19.8	2.2	5.9	7.0	8.3
	Prevotellaceae	2.1	2.0	6.3	0.0	0.0	1.7	3.7	0.5	0.7	1.9	0.0	0.1	0.3	0.5
	Propionibacteriaceae	0.7	1.4	6.2	0.0	0.0	0.1	0.6	6.0	3.2	10.3	0.2	4.1	5.6	8.8
	Rhizobiaceae	0.3	0.7	2.4	0.0	0.0	0.0	0.0	1.1	1.3	3.2	0.0	0.0	0.7	2.1
	Rhodobacteraceae	1.2	1.4	4.5	0.0	0.0	0.6	1.9	1.5	1.5	4.6	0.0	0.4	1.2	2.3
	Rikenellaceae	2.5	2.2	6.7	0.0	0.8	1.8	4.4	0.5	0.6	1.5	0.0	0.0	0.2	0.8
	Rubrobacteriaceae	0.4	0.7	2.6	0.0	0.0	0.0	0.5	1.1	2.8	9.0	0.0	0.0	0.0	0.8
	Sphingomonadaceae	11.2	10.5	31.2	0.0	2.2	7.3	18.2	14.9	6.4	25.5	6.6	10.9	13.4	18.7
	Spirosomaceae	0.8	1.4	5.8	0.0	0.0	0.0	1.1	1.7	1.8	5.3	0.0	0.4	1.2	2.2

	Streptococcaceae	4.9	7.2	22.7	0.0	0.4	2.4	4.3	1.1	1.6	4.9	0.0	0.0	0.5	1.1
Genus	*67_14*	4.7	1.4	8.8	3.2	3.7	4.4	5.4	7.6	1.5	10.5	5.6	6.5	7.4	8.0
	Agrococcus	0.8	2.0	8.2	0.0	0.0	0.0	0.6	1.7	4.6	14.7	0.0	0.0	0.2	0.6
	Anaerostipes	2.5	2.2	6.7	0.0	0.8	1.8	4.4	0.5	0.6	1.5	0.0	0.0	0.2	0.8
	Aquabacterium	1.5	1.5	4.6	0.0	0.0	1.2	2.5	0.2	0.3	0.7	0.0	0.0	0.0	0.3
	Bacteroides	0.1	0.4	1.8	0.0	0.0	0.0	0.0	2.4	2.5	7.8	0.0	1.1	1.4	4.0
	Bdellovibrio	4.8	4.3	12.4	0.0	0.7	3.5	9.8	0.9	1.2	3.1	0.0	0.0	0.5	1.3
	Bryobacter	3.0	2.4	10.3	0.3	1.4	2.6	3.6	1.3	1.6	4.9	0.0	0.1	0.9	1.8
	Cutibacterium	1.0	1.1	3.0	0.0	0.0	0.6	1.8	0.0	0.0	0.0	0.0	0.0	0.0	0.0
	Devosia	0.7	1.2	4.5	0.0	0.0	0.2	0.6	1.4	1.5	4.8	0.0	0.2	0.9	2.2
	Flavobacterium	1.4	1.8	5.9	0.0	0.0	0.1	2.2	2.7	2.1	5.3	0.0	0.6	3.1	4.4
	Hymenobacter	0.0	0.2	0.6	0.0	0.0	0.0	0.0	4.8	2.5	7.8	0.0	3.5	4.6	7.0
	JGI_0001001_H03	3.2	4.4	15.0	0.0	0.1	0.8	6.5	1.4	1.3	4.2	0.0	0.4	1.2	2.0
	Lachnospiraceae_UCG_001	2.5	1.8	5.4	0.0	1.4	2.2	4.3	0.6	1.0	2.5	0.0	0.0	0.0	0.8
	Lactococcus	6.4	10.3	37.3	0.0	0.6	3.0	5.9	2.3	3.4	9.5	0.0	0.0	0.8	2.2
	Legionella	3.9	6.6	22.4	0.0	0.0	1.0	4.1	0.8	1.6	4.9	0.0	0.0	0.0	0.6
	Massilia	0.5	1.1	4.6	0.0	0.0	0.0	0.0	3.0	4.3	13.3	0.0	0.0	1.0	3.8
	Methylobacterium	1.7	3.5	14.5	0.0	0.0	0.4	1.1	4.4	5.6	19.8	0.8	1.7	2.5	4.4
	Microlunatus	13.1	13.2	38.6	0.0	1.6	9.6	20.6	8.1	11.1	31.8	0.0	1.4	3.6	7.2
	Microvirga	0.0	0.0	0.0	0.0	0.0	0.0	0.0	1.1	1.1	2.5	0.0	0.0	1.1	1.8
	Modestobacter	0.2	0.6	2.6	0.0	0.0	0.0	0.0	1.7	1.8	4.7	0.0	0.0	1.5	2.5
	Muribaculaceae	0.7	0.7	1.8	0.0	0.0	0.9	1.3	1.6	1.6	3.7	0.0	0.0	1.8	3.1
	Mycobacterium	11.1	9.2	25.8	0.0	2.2	10.0	20.3	2.5	3.1	8.3	0.0	0.0	1.4	4.4
	Nocardioides	0.5	0.7	2.7	0.0	0.0	0.0	0.8	1.4	1.2	4.1	0.0	0.6	1.3	1.9
	Not_Assigned	0.9	2.0	8.6	0.0	0.0	0.0	1.2	3.1	3.3	9.9	0.0	0.3	2.2	4.1
	Noviherbaspirillum	12.0	4.4	18.1	5.0	7.8	12.8	15.4	23.8	10.3	37.8	9.8	15.8	23.1	31.9
	Oscillibacter	3.2	2.8	9.0	0.0	1.4	2.4	3.9	3.0	2.2	7.0	0.0	1.4	2.7	4.2
	Pseudomonas	2.1	2.0	6.3	0.0	0.0	1.7	3.7	0.5	0.7	1.9	0.0	0.1	0.3	0.5
	Rubrobacter	0.7	0.8	3.0	0.0	0.0	0.3	1.2	1.0	1.0	3.0	0.0	0.1	1.0	1.3
	Ruminococcus	0.4	0.7	2.6	0.0	0.0	0.0	0.5	1.1	2.8	9.0	0.0	0.0	0.0	0.8
	Spirosoma	9.6	9.2	27.8	0.0	2.2	5.9	16.3	8.7	4.8	17.1	3.2	5.4	7.3	12.0
	Streptococcus	0.5	1.1	4.1	0.0	0.0	0.0	0.4	1.4	1.6	4.6	0.0	0.4	0.6	1.7
